# Convergent validity of the ACC/AHA pooled cohort equations in associating with health-related quality of life among adults in the United States

**DOI:** 10.15171/hpp.2017.08

**Published:** 2016-12-18

**Authors:** Allison Nooe, Meghan K. Edwards, Ovuokerie Addoh, Paul D. Loprinzi

**Affiliations:** ^1^Center for Health Behavior Research, Physical Activity Epidemiology Laboratory, Department of Health, Exercise Science and Recreation Management, The University of Mississippi, University, MS 38677, USA; ^2^Jackson Heart Study Vanguard Center of Oxford, Center for Health Behavior Research, Physical Activity Epidemiology Laboratory, Department of Health, Exercise Science and Recreation Management, The University of Mississippi, University, MS 38677, USA

**Keywords:** Epidemiology, NHANES, Pooled cohort equations, Quality of Life

## Abstract

**Background:** The potential convergent validity of the pooled cohort risk (PCR) equations in predicting health-related quality of life (HRQOL) has yet to be evaluated, which was this study’s purpose.

**Methods:** Data from the 2001-2011 National Health and Nutrition Examination Survey (NHANES) were used (N = 8978 adults, 40-79 years, free of cardiovascular disease at baseline). Calculation of an individual’s 10-year risk of a first atherosclerotic cardiovascular disease (ASCVD) event was determined via the PCR equation. The Centers for Disease Control and Prevention (CDC) HRQOL measure was assessed utilizing 4 questions regarding participants’ perceived mental and physical health status from the past 30 days.

**Results:** When adjusting for moderate-to-vigorous physical activity (MVPA), obesity, age, gender and race-ethnicity, an ASCVD score of >20% (vs. <20%) was associated with a 0.53-unit (95% CI: 0.34-0.71) higher HRQOL score. A higher HRQOL score indicates a poorer patient perception of their mental and physical health.

**Conclusion:** The observed association between PCR-determined ASCVD-risk scores and HRQOL provides evidence for the convergent validity of the PCR algorithms, indicating that individuals with a higher risk for a first time ASCVD-event may also have an overall worse HRQOL. As such, employing ASCVD risk reduction efforts may be an important strategy in improving an individual’s HRQOL.

## Introduction

The pooled cohort risk (PCR) equations were developed by the American College of Cardiology/American Heart Association (ACC/AHA) task force in 2013 to predict 10-year risk for a first atherosclerotic cardiovascular disease (ASCVD) event.^1^ Since their development, numerous studies have employed the PCR equations, contributing evidence of independent associations between various health factors (e.g. smoking status and diabetes status) and cardiovascular disease (CVD) risk scores. Recent work by Loprinzi and Addoh provided some support for the predictive validity of the PCR equations in a longitudinal study, observing significant associations between (PCR-determined) 10-year risk of an initial ASCVD score and both CVD-specific and all-cause mortality.^2^ Other work also demonstrates evidence of these equations in predicting cancer-specific mortality.^3^

Objective measures, such as blood pressure and cholesterol, are often evaluated to measure the health status of patients, including those with CVD.^2^ Given that health encompasses not only prolonging survival, but also promoting a high quality of life throughout the entire lifespan, it is imperative to also evaluate subjective measures that may influences one’s quality of life, such as their mental health and overall physical well-being.^4^ Encouragingly, several studies have examined the association between CVD status and health-related quality of life (HRQOL). For instance, in 2014, Tan et al evaluated HRQOL (a subjective measurement to assess one’s quality of life) among type 2 diabetes mellitus (T2DM) patients with and without CVD, finding that those with CVD had significantly lower HRQOL than those free of CVD.^4^ Additionally, cardiorespiratory fitness (CRF), which is inversely associated with CVD risk,^5^ is also positively associated with HQROL parameters (e.g. perceived physical and mental health).^6^

Based on the premise of the previously mentioned associations between CVD and HQROL, the present study aimed to examine the association between PCR-determined ASCVD risk and HRQOL among a national sample of US adults. This topic (predicted CVD and HRQOL) using the PCR equations, to our knowledge, has yet to be investigated. Given that PCR equations are used to estimate 10-year risk for a first ASCVD event, and CVD is associated with HQROL, using these equations may provide clinicians with a quick and cost-effective method to not only provide diagnostic information on CVD risk, but also information related to overall HRQOL.

## Materials and Methods

### Study design **&** participants

As noted below, the PCR equations were derived among CVD-free adults ≥40 years and ≤79 years; thus, our analyses were restricted to these age ranges (i.e., 40-79 years). In the evaluated 2001-2010 National Health and Nutrition Examination Survey (NHANES) cycles, 8978 adults 40-79 years who were free of CVD constituted the analytic sample.

The NHANES is an ongoing survey conducted by the Centers for Disease Control and Prevention (CDC) that uses a representative sample of non-institutionalized US civilians selected by a complex, multistage, stratified, clustered probability design. The multistage design consists of 4 stages, including the identification of counties, segments (city blocks), random selection of households within the segments, and random selection of individuals within the households. Procedures were approved by the National Center for Health Statistics review board. Consent was obtained from all participants prior to data collection. Further information on NHANES methodology and data collection is available on the NHANES website (http://www.cdc.gov/nchs/nhanes.htm).

### PCR equations to identify 10-year ASCVD risk

Details on the derivation of the PCR equations to predict 10-year risk for a first ASCVD event are described thoroughly in the publication by the American College of Cardiology/American Heart Association (ACC/AHA) Task Force on Practice Guidelines.^7^ As described elsewhere,^8-12^ separate equations were developed for African-American and White/other men and women (four groups), which included the following variables in the equations: age (years), concentration of total cholesterol (mg/dL) and high-density lipoprotein cholesterol (HDL-C) (mg/dL), treated or untreated systolic blood pressure (mm Hg), diabetes status (defined here as physician diagnosis or A1C ≥6.5%), and self-reported smoking status (yes/no). Herein, we evaluated participants as moderate-to-high risk and high risk for a first ASCVD event based on a PCR score of ≥7.5% and ≥20% respectively.^13^

### Measurement of HRQOL

As described elsewhere,^14-19^ the CDC HRQOL measure was assessed from 4 questions, including 1 question about self-rated health status and 3 about the number of unhealthy days during the past 30 days:

1. “Would you say that in general your health is excellent, very good, good, fair, or poor?”
2. “Now thinking about your physical health, which includes physical illness and injury, how many days during the past 30 days was your physical health not good?”
3. “Now thinking about your mental health, which includes stress, depression, and problems with emotions, how many days during the past 30 days was your mental health not good?”
4. “During the past 30 days, approximately how many days did poor physical or mental health keep you from doing usual activities, such as self-care, work, or recreation?”

The 4 CDC HRQOL items were categorized according to CDC’s recommendations, which included question 1 dichotomized as good/excellent health (coded as 0) or poor/fair health (coded as 1). The latter 3 items were dichotomized as 14 or more days (coded as 1) and less than 14 days (coded as 0).

Thus, the recoded 4 HRQOL items ranged from 0-1. An overall HRQOL score was created by summing the responses from each of the 4 individual items (range: 0-4), with higher HRQOL scores indicating worse HRQOL. The HRQOL-4 developed by CDC has undergone extensive reliability and validity testing and has demonstrated adequate psychometric properties.^20-24^

### Statistical Analyses

All analyses were computed in Stata (v. 12; College Station, TX). Analyses accounted for the complex survey design employed in NHANES by utilizing sample weights, primary sampling units and strata via the Taylor series (linearization) method. A weighted ordinal regression model was used to examine the association between the PCR score on HRQOL.

Consistent with other studies,^2,3^ three models were computed, including: Model 1 being an unadjusted model; Model 2 adjusted for self-reported engagement in moderate-to-vigorous physical activity (MVPA) in the past 30 days (yes/no) and obesity (measured body mass index ≥ 30 kg/m^2^); and Model 3 adjusted for MVPA, obesity, age (years; continuous measure), gender (male/female) and race-ethnicity (Mexican American, non-Hispanic white, non-Hispanic black and other). These nested models were evaluated because the demographic parameters (age, gender and race-ethnicity) included in Model 3 were used in the PCR equations. Each of these three models were evaluated with the ASCVD score expressed as either a continuous variable, dichotomized as ≥7.5% vs. <7.5%, or dichotomized as ≥20% vs. <20%. Statistical significance was established as *P *< 0.05.

## Results

Among the 8978 CVD-free adults 40 t o 79 years, the mean (SE) age was 53.1 (0.17) years, the mean body mass index was 28.5 (0.09) kg/m^2^ and the mean PCR score was 7.17% (0.13%). In the sample, 29.9% of these CVD-free adults (95% CI, 28.6%-31.1%) had a PCR score of 7.5% or higher and 8.5% (95% CI, 7.9%-9.0%) had a PCR score of 20% or higher. In the sample, 52.3% were female and 76.2% were non-Hispanic White. These demographic characteristics are shown in Table 1. All of the provided estimates are weighted estimates which account for the complex study designed used in the NHANES. Therefore the weighted estimates are nationally representative, population-based estimates.^2^

**Table 1:** Demographic characteristics of the analyzed sample (N = 8978)

**Characteristics**	**Point estimate**
Age, mean (SE) years	53.1 (0.17)
Gender (%)	
Male	47.7
Female	52.3
Race/Ethnicity (%)	
Non-Hispanic White	76.2
Non-Hispanic Black	9.5
Other	14.3
BMI, mean (SE) kg/m^2^	28.3 (0.09)
PCR equation score, mean (SE) %	7.17 (0.13)
Adults above PCR cut-points (%)	
>7.5%	29.9
>20%	8.5

Abbreviations: PCR, pooled cohort risk; BMI, Body mass index.

Table 2 displays the weighted associations between the 10-year ASCVD risk and HRQOL. Statistically significant, positive associations (unfavorable association) between PCR-predicted ASCVD-risk and HRQOL score among all three models (unadjusted, adjusted for MVPA & obesity, and adjusted for MVPA, obesity, age, gender, and race/ethnicity) were observed. Notably, these associations were significant when PCR risk scores were analyzed as a continuous distribution and when PCR risk score was classified as a binary variable at both the 7.5% and 20% cut-points. For every 1% increase in ASCVD, there was a corresponding 0.02-unit (β = 0.018, 95% CI: 0.013-0.024, *P *< 0.05) increase in the HRQOL score as shown in Model 1, 0.01-unit (β = 0.013, 95% CI: 0.008-0.018, *P *< 0.05) increase shown in Model 2, and .03-unit (β = 0.030, 95% CI: 0.023-0.038, *P *< 0.05) increase as shown in Model 3. In cognizance of the aforementioned scoring of the HRQOL scale, a higher score is indicative of a worse HQROL. When evaluated as a binary variable, an ASCVD score of >7.5% (vs. <7.5%) was associated with a .37-unit (β = 0.37, 95% CI: 0.23-0.50, *P *< 0.05) higher HRQOL score in Model 1, a .25-unit (β = 0.25, 95% CI: 0.11-0.38, *P *< 0.05) higher score in Model 2, and a .53-unit (β = 0.53, 95% CI: 0.34-0.71, *P *< 0.05) higher score in Model 3. Those with an ASCVD score of >20% or higher (vs. <20%) had a .48-unit (β = 0.48, 95% CI: 0.32-0.64, *P *< 0.05) higher HRQOL score in Model 1, 0.36-unit (β = 0.36, 95% CI: 0.21-0.52, *P *< 0.05) higher HRQOL score in Model 2, and 0.51-unit (β = 0.51, 95% CI: 0.32-0.70, *P *< 0.05) higher HRQOL score in Model 3.

**Table 2:** Weighted association between 10-year ASCVD risk and health-related quality of life using the pooled cohort equations, 2001-2010 National Health and Nutrition Examination Survey (N = 8978).

	**β (95% CI)**		
	**Model 1 (unadjusted)**	**Model 2 (MVPA and obesity adjusted)**	**Model 3 (MVPA, obesity, age, gender and race-ethnicity adjusted)**
ASCVD risk score, 1% increase	**0.018 (0.013-0.024)**	**0.013 (0.008-0.018)**	**0.030 (0.023-0.038)**
ASCVD risk score, ≥7.5% vs. <7.5%	**0.37 (0.23-0.50)**	**0.25 (0.11-0.38)**	**0.53 (0.34-0.71)**
ASCVD risk score, ≥20% vs. <20%	**0.48 (0.32-0.64)**	**0.36 (0.21-0.52)**	**0.51 (0.32-0.70)**

Abbreviations: ASCVD, atherosclerotic cardiovascular disease; MVPA, moderate-to-vigorous physical activity. Bold indicates statistical significance (*P *< 0.05).

## Discussion

The purpose of this study was to evaluate the convergent validity of the PCR equations in associating with HRQOL. The primary finding among this nationally representative sample of US adults was an observed association between PCR-determined ASCVD risk and HQROL. For example, when looking specifically at Model 3, an ASCVD score of >20% (vs. <20%) was associated with a .53-unit higher HRQOL score (indicating poorer patient-perceived mental and physical health).

This association between PCR score among adults free of CVD and HQROL suggests that, even in the absence of CVD, individuals with higher CVD-risk seem to have a relatively lower HRQOL. These findings are in alignment with previous work showing associations between PCR-determined ASCVD-risk and various health outcomes, as well as evidence suggesting CVD status is associated with HRQOL. The variables examined in the PCR equations (age, smoking and diabetes status, total concentrated cholesterol levels, and blood pressure) have all been shown to associate with HRQOL. For example, a study by Wang et al found that out of over 900 participants, those with hypertension had poorer HRQOL scores than those without, also noting that the dimensions most affected were those regarding roles of physical limitations.^25^ Other research has also corroborated this worse HRQOL among those with hypertension.^26^ Hypertension, in particular, may induce worse HRQOL via its potential effects on both physical^27^ and mental health.^28^ A review study by Rubin and Peyrot concluded that most studies demonstrated that HRQOL was lower among those with diabetes when compared to the general population.^29^ This is an expected finding given that diabetes is also associated with parameters (e.g., muscle strength) associated with physical function.^30^ Diabetes may also influence mental health via its putative effect on inflammation.^31^ Thus, our observed association of the collective contributions of the parameters assessed in the PCR equations with HRQOL aligns with previous research that has evaluated the effects of these individual health parameters on HRQOL.

In conclusion, our findings demonstrate that those with a higher predicted score of a first ASCVD event within the next 10 years had worse HRQOL. A study by Loprinzi and Addoh^2^ demonstrated a positive association between PCR-determined 10-year risk of a first ASCVD event and all cause/CVD-specific mortality, as well as cancer-specific mortality.^3^ Thus, early identification of CVD risk, which may be achieved using these equations, may elicit earlier intervention approaches and thus a greater opportunity to enhance HRQOL among US adults. A limitation of this study is the cross-sectional study design. Notable strengths include the nationally representative sample and the novelty of the subject matter. Future studies may further examine the directionality of our observed association between ASCVD risk and HRQOL using a longitudinal study design.

## Ethical approval

This study was approved by the ethics committee of the National Center for Health Statistics.

## Competing interests

We declare no conflicts of interest.

## Authors’ contributions

All authors were involved in the conceptualization of the study, revising the manuscript and interpreting the results. Author AN drafted the first draft of the manuscript and author PDL computed the analyses.
